# Oral Health Care in Hong Kong

**DOI:** 10.3390/healthcare6020045

**Published:** 2018-05-11

**Authors:** Sherry Shiqian Gao, Kitty Jieyi Chen, Duangporn Duangthip, Edward Chin Man Lo, Chun Hung Chu

**Affiliations:** Faculty of Dentistry, The University of Hong Kong, Hong Kong, China; kjychen@hku.hk (K.J.C.); u3001284@connect.hku.hk (D.D.); hrdplcm@hku.hk (E.C.M.L.); chchu@hku.hk (C.H.C.)

**Keywords:** health care, oral health, dental caries, water fluoridation, dental education, Hong Kong

## Abstract

Hong Kong, as a special administrative region of the People’s Republic of China, is a metropolitan city in Asia with a population of approximately 7.4 million. This paper reflects the oral health care situation in Hong Kong. Water fluoridation was introduced in 1961 as the primary strategy for the prevention of dental caries. The fluoride level is currently 0.5 parts per million. Dental care is mainly provided by private dentists. The government’s dentists primarily serve civil servants and their dependents, with limited emergency dental service for pain relief offered to the general public. Nevertheless, the government runs the school dental care service, which provides dental treatments to primary school children through dental therapists. They also set up an oral health education unit to promote oral health in the community. Hong Kong had 2280 registered dentists in 2017, and the dentist-to-population ratio was about 1:3200. The Faculty of Dentistry at the University of Hong Kong is the only institution to provide basic and advanced dentistry training programs in Hong Kong. Dental hygienists, dental surgery assistants, dental therapists, and dental technicians receive training as paradental staff through the university or the government.

## 1. Introduction

Hong Kong is a metropolitan city located on the southeast coast of the People’s Republic of China. It consists of 262 islands spanning a total area of 1054 square kilometers. As one of the special administrative regions of the People’s Republic of China, Hong Kong enjoys a ‘high degree of autonomy’ under the ‘one country, two systems’ policy. It presents high international rankings in various aspects, such as the Human Development Index, quality of life, financial and economic competitiveness, economic freedom, and corruption perception. The Hong Kong dollar (HK$) has been pegged to the United States dollar (US$) since 1983, and the currency exchange rate is currently around US$ 1 = HK$ 7.8. In 2013, the per-capita gross domestic product (GDP) was about US$ 38,000 in Hong Kong, and the total expenditure on health was about 5.7% of GDP [1].

The population in Hong Kong steadily increased to around 7.4 million in 2017 [2]. The large majority of the population is Southern Chinese (91%), while Filipinos and Indonesians make up 5% of the population [3]. Similar to other countries, the aging of the population is an important demographic issue in Hong Kong. The proportion of elderly people aged 65 years or older was 17% in 2017, and is expected to rise to 27% in 2033. Around 11% of the population are below 15 years old, and 32% are 45 to 64 years old. The median age of the entire population was 42 in 2017 [4]. In addition, the mortality rate in 2016 was 6.4 per 1000 within the entire population, which is one of the lowest in the world. Similar to the situations in other developed countries, the infant mortality rate (1.5 per 1000 live births in 2015) and maternal mortality rate (1.6 per 100,000 live births in 2015) have decreased within the past few years [5]. Hong Kong also presents the highest life expectancy worldwide. The average life expectancy of males and females is 81.3 and 87.3 years, respectively [6]. Moreover, the number of medical doctors in Hong Kong is approximately 14,000, with the doctor-to-population ratio being around 1:520 [7].

## 2. Water Fluoridation

The domestic water supply can reach nearly the entire area of Hong Kong, including the rural parts and major remote islands. Water fluoridation was implemented in 1961 in Hong Kong and is considered the most significant strategy for the primary prevention of dental caries. The initial fluoride level was at 0.8 parts per million (ppm). In 1967, it was increased to 1.0 ppm. However, a survey found that a fluoride concentration of 1.0 ppm is excessive, and thus can lead to a high prevalence of dental fluorosis among 7- to 12-year-old children [8]. Dental fluorosis is an unaesthetic tooth defect caused by excessive intake of fluoride during enamel formation, which can result in the hypomineralization of tooth enamel. Therefore, in 1978, the fluoride concentration was reduced to 0.7 ppm, and in 1988, it was further adjusted to 0.5 ppm [8]. Dental fluorosis decreased accordingly because of the reduction of the fluoride level in water [9].

Since water fluoridation has been implemented, it has been beneficial to the local population’s dental health [10]. In 1960, the caries experience in the mean decayed, missing (due to caries), and filled teeth for permanent teeth (DMFT) among 7- to 12-year-old Hong Kong children was 4.4 [8]. After water fluoridation was introduced, the mean DMFT was decreased to 1.5 in 1968. Further reductions were observed in 2001 (DMFT = 0.8) and 2011 (DMFT = 0.4) among 12-year-old children [11].

## 3. Oral Health Conditions

The prevalence of dental caries among 12-year-old children in Hong Kong was 23% in 2011. The caries experience in the mean DMFT was 0.4, which was the lowest worldwide [11]. Although the caries experience among 12-year-old children has decreased over the years, the dental caries status among preschoolers is unsatisfactory. A survey in 2017 reported that more than half of 5-year-old preschool children suffered from dental caries, and most of the caries were left untreated [12]. The caries experience in the mean decayed, missing (due to caries), and filled teeth for primary teeth was 2.7 [12]. The caries experience among the adult population was also considerable in Hong Kong. The mean DMFT of middle-aged people (35–44 years old) was 6.9, and 4% of them had root caries. Meanwhile, the elderly population (65–74 years old) presented a mean DMFT of 16.2, and 25% of them had root caries [11].

The periodontal condition deteriorated with age among Hong Kong people [11]. Most 12-year-old children (86%) had gingivitis. Calculus was prevalent (64%) among them [11]. Moving to the adults, only 1% of 35- to 44-year-old people had healthy gums. Eighty percent of them had bleeding gums surrounding at least half of their teeth, and 40% of them had deep pockets. In the elderly population (over 65 years old), it was found that almost all (97%) of them had bleeding gums, and more than half (60%) presented deep pockets [11]. Hence, the periodontal status indicates that people in Hong Kong need professional periodontal care, such as scaling and the polishing of teeth.

The incidence of oral cancer in Hong Kong was 5.5 per 100,000 among females and 12.2 per 100,000 among males in 2015. In addition, the mortality rate is 1.5 per 100,000 and 4.6 per 100,000 in females and males, respectively [13]. It is noteworthy that 18.6% of the males were daily cigarette smokers, whereas only 3.2% of the females were daily smokers [14]. In addition, the proportion of current drinkers in males was as twice as high as that in females [15]. The smoking and drinking situations may relate to the different incidences of oral cancer among males and females. Similar to the situations of other populations, squamous cell carcinoma is the most prevalent oral cancer in Hong Kong. The fundamental treatment of oral cancer is surgery followed by radiation therapy, which the government highly subsidizes. Oral complications, such as dry mouth and radiation caries, are common in patients undergoing long-term radiation therapy. Therefore, these patients need follow-up oral care to improve their quality of life.

## 4. Oral Health Practices

Daily oral health practices highly influence an individual’s oral health condition. In Hong Kong, 95% of the population practice tooth brushing everyday [11]. Fluoridated toothpastes are common in the market. However, the majority of the population use toothpicks, rather than dental floss, for interdental cleaning. Although products for oral health care are widely available and accessible in Hong Kong, people’s awareness and knowledge of oral health remains low. As a result, many people in Hong Kong consider dental diseases to be a low priority [10]. A survey conducted in Hong Kong reported that 73% of the respondents did not know about the signs and symptoms of dental erosion, and more than half of the participants (53%) could not distinguish dental erosion from dental caries [16].

## 5. Oral Health Care Systems

Dental service is mainly provided through the private sector in Hong Kong. Most of the dentists are working alone or under small groups of general practitioners. No regulation exists regarding the consultation and treatment fees that the government sets. Thus, huge variations in the services provided and fees charged may be observed within various clinics. Nevertheless, the Dental Council of Hong Kong has issued strict ethical guidelines for the advertisement of dental practices [17].

Apart from private practices, around 60 nongovernmental organizations (NGOs) in Hong Kong provide dental services. These NGOs are usually welfare organizations, religious groups, labor unions, or social service agencies. Basically, they do not receive subsidies from the government. Therefore, dental services are self-financed and/or covered with donations. Many NGO dental clinics provide similar dental services to those of private practices, but at a relatively lower price. The target population is usually people with low socioeconomic statuses or those from disadvantaged groups, such as the elderly and physically handicapped.

The government dental clinics that the department of health runs offer dental services mainly to civil servants, the dependents of civil servants, and civil servant pensioners. Limited emergency dental services for pain relief, such as tooth extractions, are provided to the public. The oral maxillofacial surgery and dental units under the department of health also provide emergency and specialist dental services, but only for referred patients or hospitalized patients with special oral health care needs.

Although the government provides limited dental care services, the department of health set up a dental health program for Hong Kong primary school children, named the School Dental Care Service (SDCS) [18]. This program was established in 1979 and is now running in nine SDCS clinics. It provides oral health education, dental examinations, preventive and basic restorative treatments for dental caries, and emergency dental services to children; however, orthodontic treatment and other advanced dental treatments are excluded. Professionally trained dental therapists provide these services under the supervision of government dentists. All primary school children (mostly aged 6–12 years) are eligible for SDCS, and the enrollment is voluntary through schools. A nominal enrollment fee of around US$ 2.50 (HK$ 20) per child is charged each year and covers all of the dental services that the child receives.

The department of health has also executed a health care voucher scheme for elderly individuals since 2008 [19]. This scheme is available for 65- to 69-year-old individuals who are eligible to apply for the Normal Old Age Allowance. Currently, this scheme provides 10 vouchers of around US$ 6 (HK$ 50) per entitled elderly individual annually. The vouchers can be used to cover dental costs in private clinics. Although the majority of elderly individuals appreciate this scheme, many of them still consider US$ 60 (HK$ 500) per year to be insufficient to subsidize their dental care expenses.

The department of health established an oral health education unit for oral health promotion to the general public [20]. The unit has been organizing various activities to promote oral health. Amongst them, the ‘Love Teeth Campaign’ is one of the most well-known oral health events in Hong Kong. This campaign was founded in 2011 [21]. Its aim is to raise awareness about oral health and to deliver information on preventing oral diseases. Related events are held every year, including lectures, consultation services, games, and oral health education for children. Apart from these events, oral-health-related messages are disseminated through local television, internet, radio, and newspaper advertisements, complemented by posters and banners displayed in public venues. The ‘Love Teeth Campaign’ is also conjoined with ‘World Oral Health Day’ to bring more international attention to the local event.

The oral health education unit has developed a dedicated website called ‘Tooth Club’ to disseminate information about oral health care through the internet to the general public [20]. The oral health education unit also distributes free materials for promoting oral health, such as pamphlets, brochures, posters, and video compact discs, to local schools and organizations from time to time. In addition, local schools and organizations can borrow various oral health education materials from the unit, such as games, models, and exhibits. The oral health education unit also provides a program called ‘Brighter Smiles Playland’, which is specially designed for 4-year-old children in local kindergartens and nursery schools. Through this program, children are expected to obtain and absorb oral health-related knowledge in an interactive way.

## 6. Dentist Profile

Dentists working in Hong Kong were all trained overseas before 1985. In 1980, under the requirements set by the General Dental Council of the United Kingdom, the Dental Studies at the University of Hong Kong (HKU, Hong Kong, China) admitted its first batch of dental students. After a five-year training course, 70 dental students graduated with Bachelor of Dental Surgery (BDS) degrees and began to practice in 1985. In 1982, the HKU Faculty of Dentistry was established and housed at the Prince Philip Dental Hospital (PPDH). So far, the HKU Faculty of Dentistry remains the only institution in Hong Kong that provides dental education. Since 2012, the training duration for BDS has been extended to six years. Completing a license examination prior to practicing is not required for those who graduated with BDS degrees from the HKU Faculty of Dentistry. Currently, around 70 BDS students are enrolled every year. More than 1600 dentists had trained under the HKU Faculty of Dentistry by April 2017. Dental graduates who have trained abroad can also practice dentistry in Hong Kong when they pass the license examination that the Dental Council of Hong Kong has established. Hong Kong had approximately 2280 registered dentists in 2017, and the dentist-to-population ratio was approximately 1:3200. In addition, approximately three-quarters of dentists practice in private dental clinics. Meanwhile, approximately one-fifth are enrolled in the government sectors. Dentists also work in NGOs or the HKU Faculty of Dentistry, or join the available postgraduate training program.

Apart from the undergraduate training, the HKU Faculty of Dentistry offers postgraduate clinical training in various dental specialties [22]. The College of Dental Surgeons of Hong Kong was established in 1993, which aimed to manage and promote postgraduate dental training and dental research. In Hong Kong, eight recognized specialties exist, including community dentistry, endodontics, family dentistry, oral and maxillofacial surgery, orthodontics, pediatric dentistry, periodontology, and prosthodontics [23]. Being a dental specialist requires at least six years of advanced training, which include a three-year master’s degree (basic training) and three years of supervised specialty-related clinical practice (higher training). In 2017, the majority of dentists in Hong Kong were general practitioners, whereas around 12% of them were registered dental specialists.

## 7. Paradental Staff

Apart from dentists, paradental staff also work as an essential part of the dental team, which includes dental hygienists, dental surgery assistants, dental therapists, and dental technicians.

Dental hygienists are enrolled as professionals in Hong Kong. Their responsibilities include completing dental examinations; cleaning, scaling, and polishing of teeth; taking oral radiographs; applying topical fluorides and sealants; and providing oral health education. However, a dentist must have diagnosed the patient before a dental hygienist provides a service to him or her. In addition, a dentist must be available at all times on the premises when the dental hygienist is providing the prescribed dental procedures. A total of 424 dental hygienists were enrolled in 2016, and 210 of them were practicing [7]. A survey revealed that more than 90% of dental hygienists were working in the private sector in 2014 [24]. The PPDH began to offer a one-year training program for dental hygienists in the early 1980s. Since 2002, the program has been expanded to a two-year Higher Diploma in Dental Hygiene, which the Community College of HKU and the PPDH conjointly oversee. 

Dental surgery assistants work closely with dentists in the clinic to provide dental services to patients. They are essential team members and are indispensable participants in four-handed operations in dental practices. The responsibilities of dental surgery assistants are not limited to chair side assisting; rather, they also complete appointment booking, receive patients, receive payments, maintain stock, provide oral health education, and sterilize instruments. Around 3800 dental surgery assistants were enumerated in 2014, and more than 85% of them worked in private dental clinics [24]. Dental surgery assistants in Hong Kong are not required to be enrolled. The Hong Kong Polytechnics provided a two-year full-time diploma program for dental surgery assistant training that started in 1980, but the program was discontinued in 1984. The department of health once trained dental surgery assistants through an in-service program, but it was also discontinued in the 1980s. So far, dental surgery assistant training programs are not standardized in Hong Kong. Various training courses are available in the market. The PPDH provides one-year full-time or two-year part-time certificate programs for dental surgery assistant training. Apart from these, commercial health institutions also offer training to dental surgery assistants. In addition, private dentists can hire lay persons for on-the-job training.

The department of health employs dental therapists, who also are not required to be enrolled. Most of them work in the SDCS. Their responsibilities include completing oral and radiographic examinations, providing preventive treatment (scaling, the application of topical fluoride and fissure sealants), providing basic dental treatment (fillings and extraction), and offering oral health education. All of the procedures that dental therapists perform should be completed under the supervision of a dentist. As of 2014, a total of 284 dental therapists were enumerated in Hong Kong [24]. Dental therapists were trained in a three-year in-service training program through the department of health starting in 1977. However, the training of dental therapists was later suspended because of the decrease in the birth rate from 2002 to 2015. However, in 2016, the training program was restarted and transformed into a one-year Advanced Diploma in Dental Therapy, held in the PPDH. Dental hygienists with diplomas are eligible to be enrolled in this program. Ten students are recruited every year.

Dental technicians (technologists) work mainly in dental manufactories or laboratories and are not required to be enrolled. As of 2014, more than 350 dental technicians were enumerated, and most of them (77%) worked in the private sector [24]. Dental technicians are responsible for manufacturing removable and fixed dental appliances, such as complete and partial dentures, inlays, crowns and bridges, orthodontic appliances, and oral and maxillofacial prostheses. The Hong Kong Polytechnics first provided a three-year, full-time diploma in dental technology in the late 1970s. Then, the training program was relocated to the PPDH in the 1980s. The PPDH now offers a two-year full-time Advanced Diploma in Dental Technology program for dental technicians. The technicians only work in the laboratory and provide no clinical care. This is different from other countries such as the United States and Canada (which have denturist), the United Kingdom (which has clinical dental technician), and Australia (which has dental prosthetist).

## 8. Discussion

The policy of the Hong Kong government on dental services is to raise public awareness of oral health and to encourage proper oral health habits through promotion and education. The government also set up SDCS in 1979 to provide basic and preventive dental care to primary school students. SDCS is a demonstration of a successful dental health program in Hong Kong. More than 95% of Hong Kong school children aged 6–12 years are joining SDCS. The caries experience of 12-year-old children gradually decreased after the introduction of the program [25]. In 2011, 12-year-old Hong Kong children enjoyed the lowest caries experience (DMFT = 0.4) worldwide, and most of the decayed teeth were restored (filled teeth, FT = 0.3) [11]. The dental care of SDCS is largely provided by dental therapists. Dental therapists, who used to be named dental nurses, were first proposed in New Zealand to address the high dental demands of children and the shortage of dentists [26]. The Hong Kong government had adopted this model from New Zealand by sending people there to receive training as dental therapist in the 1970s. Until recently, more than 53 countries have adopted the dental therapist model in their dental health care system to address dental needs. More than 14,000 dental therapists are presently working worldwide to provide dental care to the public [27]. There used to be a debate on whether the implementation of dental therapists improved the access to dental care or affected the quality of dental care. Nevertheless, reviews reported that the treatments provided by dental therapists were technically competent, safe, and effective, especially for children [28]. As for Hong Kong, the SDCS contributes to the low caries experience of the school children [11]. Thus, the adoption of dental therapists can be a promising strategy to lower the social burden of dental care and improve the oral health of the people under served.

Moreover, the government set up the oral health education unit to deliver oral health education to the public. The unit introduced the ‘Brighter Smiles for the New Generation’ program in 1993 to promote oral health for preschool children. This program also provides dental education to increase oral health care knowledge of kindergarten teachers and parents of the preschool children. Dental care service is mainly provided by private practitioners in Hong Kong. Because of the low dentist-to-population ratio and the limited available resources, more than half (51%) of Hong Kong preschoolers had early childhood caries experience. Furthermore, most of the affected children have never seen a dentist and the carious teeth were left untreated [12]. To address this severe problem, NGOs and the HKU Faculty of Dentistry have pioneered outreach dental care programs to manage dental caries using the atraumatic restorative technique and silver diamine fluoride therapy.

It is noteworthy that in the Hong Kong adult population, the number of decayed teeth presented as the smallest component in the DMFT score (0.7/6.9 or 10% in 35- to 44-year-olds and 1.3/16.2 or 8% in 65- to 74-year-olds), whereas the number of missing teeth due to caries presented as the highest component (3.4/6.9 or 49% in 35- to 44-year-olds and 12.7/16.2 or 78% in 65- to 74-year-olds) [11]. This might be a result of patients only seeking treatment when their affected teeth were in advanced disease stages, or because the patients’ attitude to save and keep their teeth was poor.

## 9. Conclusions

Water fluoridation is the primary strategy for the prevention of dental caries in Hong Kong. The dentist-to-population ratio was around 1:3200 in 2017. Most dentists work as private practitioners. The department of health has established various programs and activities to promote oral health among different populations. Only one university in Hong Kong provides basic and advanced training programs in dentistry. Paradental staff members, such as dental hygienists, dental surgery assistants, dental therapists, and dental technicians, also receive training through the university or the government.
